# Radiative MHD thin film flow of Williamson fluid over an unsteady permeable stretching sheet

**DOI:** 10.1016/j.heliyon.2018.e00825

**Published:** 2018-10-10

**Authors:** Zahir Shah, Ebenezer Bonyah, Saeed Islam, Waris Khan, Mohammad Ishaq

**Affiliations:** a Department of Mathematics, Abdul Wali Khan University, Mardan, Khyber Pakhtunkhwa 23200, Pakistan; b Department of Information Technology Education, University of Education Winneba-(Kumasi Campus), Kumasi 00233, Ghana; c Department of Mathematics, Islamia College University, Peshawar, Khyber Pakhtunkhwa 25000, Pakistan

**Keywords:** Applied mathematics, Computational mathematics

## Abstract

In this research work we have examined the flow of Williamson liquid film fluid with heat transmission and having the impact of thermal radiation embedded in a permeable medium over a time dependent stretching surface. The fluid flow of liquid films is assumed in two dimensions. By using suitable similarity transformation the governing non-linear partial differential equations have been transformed into non-linear differential equations. An optimal approach has been used to acquire the solution of the modelled problem. The convergence of the technique has been shown numerically. The impact of the Skin friction and Nusslet number and their influence on thin film flow are shown numerically. Thermal radiation, unsteadiness effect and porosity have mainly focused in this paper. Furthermore, for conception and physical demonstration the entrenched parameters, like porosity parameter k, Prandtl number Pr, unsteadiness parameter S, Radiation parameter Rd, Magnetic parameter M, and Williamson fluid parameter have been discussed graphically in detail with their effect on liquid film flow.

## 1 Introduction

The flow analysis of thin film has got important loyalty due to its enormous applications in the field of engineering and technology in several years. The field of thin film flow problems is vast and is realized in many fields, starting from the particular situation of the flow in human lungs to lubricant problems in industry. Investigating the uses of thin liquid film flow is an interesting interaction between structural mechanics, fluid mechanics, and theology. Extrusion of polymer and metal, striating of foodstuff, constant forming, elastic sheets drawing, and fluidization of the devices, exchanges, and chemical treating apparatus are several well-known uses of liquid films. In observations of these uses and applications, the study of liquid film becomes necessary for researchers to further investigate and make further development in it. Different approaches with modified geometries have been adopted by many researchers from time to time. In view of the industrial applications of thin film flow, stretching surface has become an important topic for researchers. In early days, the study of liquid film flow was limited to viscous fluids. Crane [1] is the pioneer to deliberate the flow of viscid fluid in a linear extending surface. Dandapat [2] has deliberate viscoelastic fluid flow on an extending surface with heat transfer. Wang [3] was the first one to investigate finite liquid film at a time depending stretched surface. Ushah and Sridharan [4] have investigated the flow of finite thin liquid over a time depending stretching surface. The same work is extended by Liu and Andersson [5] using numerical techniques. Aziz et al. [6] has examined the consequence of inner heat production on flow in a thin liquid film on a time depending stretching sheet. Recently, Tawade et al. [7] has reviewed the liquid flow over an unstable extending sheet with thermal radioactivity. Andersson [8] is the forerunner to investigate the flow of tinny liquid films of non-Newtonian fluids in an unsteady stretching sheet by considering the Power law model. Waris et al. [9] has studied the nanoliquid film flow over an unstable stretching sheet with varying viscosity and thermal conductivity. Andersson et al. [10], Chen [11, 12], and Wang et al. [13], have deliberated thin liquids flow using different physical configuration. Singh Megahe et al. [14] has examined tinny film flow of Casson fluid in the occurrence of irregular heat flux and viscid dissipation. Abolbashari et al. [15] work out thin film flow with entropy generation. Qasim et al. [16] has studied the Nano fluid thin film on an unstable extending surface taking Buongiorno's model.

Non-Newtonian fluids have so many types in nature as well as in artificial. Williamson fluid is one of significant subtypes between them. A number of researchers investigated Williamson fluid with different effects. Practical application has produced interest in searching the solvability of differential equation governing in flow of Non-Newtonian liquids, which have numerous uses in engineering field, applied mathematics and computer science. Many environmental and industrial systems like system of geothermal energy and system of heat exchanger design include the convection flow subject to permeable medium. The adapted form of classical Darcian model is the non-Darcian porous medium, which contains the inertia and boundary topographies. The standard Darcy's law is effective under constrained range of small permeability and little velocity. Forchheimer [17] has predicted the inertia and boundary features by including a square velocity term to the countenance of Darcian velocity. Muskat [18] has entitled this term as “Forchheimer term” which is permanently operative for large Reynolds number. Dawer et al. [19] have studied fluid flow in porous media. The more current investigational and theoretical study of Sheikholeslami [20, 21, 22] on nanofluids using dissimilar phenomena, with modern application, possessions and properties with usages of diverse approaches can be studied in Tahir et al. [23] have studied flow of a nano-liquid film of maxwell fluid with thermal radiation and magneto hydrodynamic properties on an unstable stretching sheet. The stuided and application of porous media can been seen in [24, 25].

In (1992) Liao [26, 27] was the first one to investigate Homotopy Analysis method. Due to its fast convergence, many researchers Shah et al. [28, 29, 30, 31], Ishaq et al. [32], Saleem et al [33]. Hameede and Muhammad et al. [34, 35] have used this method to answer highly non-linear combined equations. Khan et al. [36, 37] have used this method for the solution of Boundary layer flow problems. Prasannakumara et al. [38] investigated Williamson nanofluid with impact of chemical reaction and nonlinear radiation embedded in a permeable sheet. Krishnamurthy et al. [39] have investigates slip flow and heat transmission of nanofluid over a porous stretching sheet with impact of nonlinear thermal radiation. Chaudhary et al. [40] has explored thermal radiation properties of fluid on the extending stretching surface. Das [41] has studied properties of thermophoresis and thermal radiation convective flow with heat transmission analysis. Muhammad et al. [35] have examined radiative flow of MHD carbon nanotubes. The more recent study about thermal radiation and can be studied in [42, 43].

In all of the discussed work, researchers consider heat and mass transmission features of Newtonian or non-Newtonian fluid at a time depended and a time independent extending surface, taking one or more physical characteristics. The main goal of this research is to investigate liquid film flow Williamson fluids over a stretched surface in the existence of magnetic field and thermal radiation. Keeping in view all these assumptions taken into the modelled problem and the similarity transformation method, the concerned PDEs are converted to non-linear ODEs, and the obtained, transformed equations are analytically solved using HAM.

## 2 Theory/Calculation

**Fig. 1:**
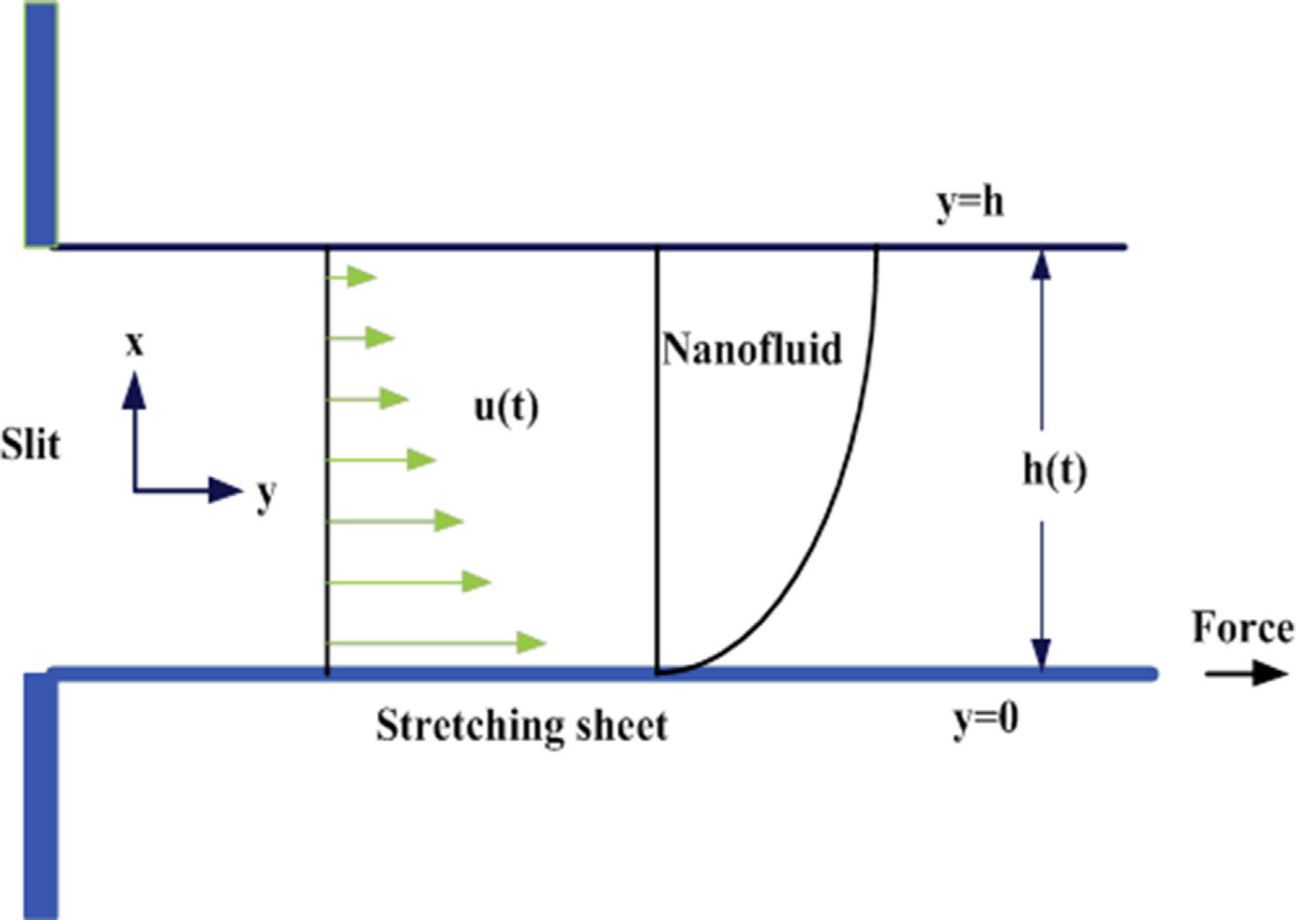
Geometry of the demonstrated problems.

Consider the flow of non-Newtonian liquid film flow (considering Williamson fluid) with impact of thermal radiation over an unsteady porous stretching sheet. The coordinate system is chosen in such a way that the x-axis is parallel to the slit while the y-axis is perpendicular to the surface respectively (Fig. 1). The x-axis is taken along the spreading surface with stress velocity as $U_{0}\left(x,\text{t}\right)=\frac{\varepsilon x}{1-\xi \text{t}}\text{,}$ where ξ≻0, is the stretching parameter. The heat transmission to the fluid flow and the temperature is defined as ${\text{T}}_{\text{s}}\left(x,\text{t}\right)={\text{T}}_{\circ }-{\text{T}}_{ref}\left[\frac{\xi x^{2}}{2\upsilon }\right]{\left(1-\varepsilon \text{t}\right)}^{-1.5}\text{,}$ called surface temperature fluctuating with the distance x from the slit. The time dependent term $\frac{\varepsilon x^{2}}{\upsilon \left(1-\xi t\right)}$ can be renowned as the local Reynold number, reliant on the velocity $U_{0}\left(x,t\right)$. Here $T_{0}$ is temperature at the slit, $T_{ref}$ is the reference temperature such that $0\leq {\text{T}}_{ref}\leq {\text{T}}_{0}$. The slit is fixed at the origin initially and then some exterior force is acting to stretch the slit at the rate $\frac{\varepsilon x}{1-\xi \text{t}}$ in time 0≤ξ≤1 with velocity $U_{0}\left(x,t\right)$ is in the positive *x*-direction. Also ${\text{T}}_{s}\left(x,t\right)$ designates the sheet temperature, reduce from ${\text{T}}_{0}$ at the slit in 0≤ξ≤1.

In the interpretation of above expectations, the main leading equations are articulated as:

(1) $$\frac{\partial u}{\partial x}+\frac{\partial v}{\partial y}=0\text{,}$$

(2) $$\left(\frac{\partial u}{\partial t}\right)+u\left(\frac{\partial u}{\partial x}\right)+v\left(\frac{\partial u}{\partial y}\right)=\frac{\mu_{0}}{\rho }\left[\left(\frac{\partial^{2}u}{\partial y^{2}}\right)+\sqrt{2}\Gamma \left(\frac{\partial u}{\partial y}\frac{\partial^{2}u}{\partial y^{2}}\right)\right]-\left(\frac{\upsilon \phi }{\rho k}-\frac{\sigma B_{0}^{2}}{\rho }\right)u\left(t\right)\text{,}$$

Here in Eqs. (1) and (2) υ represents the kinematics viscosity where $\upsilon =\frac{\mu_{0}}{\rho }$, Γ>0 represents the material constant of the Williamson fluid, ρ is the density of the fluid and σ denotes the electrical conductivity.

(3) $$\left(\frac{\partial T}{\partial t}\right)+u\left(\frac{\partial T}{\partial x}\right)+v\left(\frac{\partial T}{\partial y}\right)=\frac{k}{\rho C_{p}}\left(\frac{\partial^{2}T}{\partial y^{2}}\right)-\frac{1}{\rho C_{p}}\left(\frac{\partial q_{r}}{\partial y}\right)\text{.}$$

Here $q_{r}$ is Rosseland approximation of the radioactive heat flux and is modelled as,

(4) $$\frac{\partial q_{r}}{\partial y}=-\frac{4\sigma^{*}}{3k^{*}}\frac{\partial \left(T^{4}\right)}{\partial y}\text{,}$$

Here T represents the temperature fields, $\sigma^{*}$ is the Stefan-Boltzmann constant, $K^{*}$ is the mean absorption coefficient, k is the thermal conductivity of the liquid film. Expanding $T^{4}$ using Taylor's series about $T_{0}$ as below

(5) $${\text{T}}^{4}={\text{T}}_{0}^{4}+4{\text{T}}_{0}^{3}\left(\text{T}-{\text{T}}_{0}\right)+6{\text{T}}_{0}^{2}{\left(\text{T}-{\text{T}}_{0}\right)}^{2}+.\ldots \text{,}$$

Neglecting the higher order terms Eq. (5)

(6) $${\text{T}}^{4}≅-3{\text{T}}_{0}^{4}+4{\text{T}}_{0}^{3}\text{T}\text{,}$$

Inserting Eq. (6) in Eq. (4) we obtain

(7) $$\frac{\partial q_{r}}{\partial y}=-\frac{16{\text{T}}_{\infty }^{*}\sigma^{*}}{3k^{*}K^{*}}\frac{\partial^{2}{\text{T}}^{4}}{\partial y^{2}},$$

By putting Eq. (7) in Eq. (4), it reduced as

(8) $$\frac{\partial \text{T}}{\partial t}+u\frac{\partial \text{T}}{\partial x}+v\frac{\partial \text{T}}{\partial y}=\frac{k}{\rho C_{p}}\frac{\partial^{2}\text{T}}{\partial y^{2}}-\frac{1}{\rho C_{p}}\left(\frac{16{\text{T}}_{\infty }^{*}\sigma^{*}}{3K^{*}}\frac{\partial^{2}{\text{T}}^{4}}{\partial y^{2}}\right)\text{,}$$

The accompanying boundary conditions here in Eqs. (1) and (2)

(9) $$\begin{array}{l} u=U,\;v=0,\;T=T_{s},\;\text{at}\;\text{y}=\text{0,}\\ \frac{\partial u}{\partial y}=\frac{\partial T}{\partial y}=0\;\text{at}\;\text{y}=\text{h.,} \end{array}$$

Familiarizing the dimensionless (f) variables and similarity transformations (η) to reduce Eqs. (2), (8), and (9)

(10) $$\begin{array}{l} f\left(\eta \right)=f\left(\eta \right)=\psi \left(x,y,t\right){\left(\frac{vb}{1-at}\right)}^{-\frac{1}{2}},\;\eta =\sqrt{\frac{b}{\upsilon \left(1-at\right)}}y,\;h\left(t\right)=\sqrt{\frac{\upsilon \left(1-at\right)}{b}}\text{,}\\ \theta \left(\eta \right)=T_{0}-T\left(x,y,t\right){\left(\frac{bx^{2}}{2v{\left(1-at\right)}^{-\frac{3}{2}}}\left(T_{ref}\right)\right)}^{-1} \end{array}$$

The stream function ψ(x,y,t) satisfying Eq. (1), and in term of velocity components is obtained as

(11) $$u=\frac{\partial \psi }{\partial y}=\frac{bx}{\left(1-at\right)}f^{\prime }\left(\eta \right),v=-\frac{\partial \psi }{\partial x}=-{\left[\frac{\upsilon b}{1-at}\right]}^{\frac{1}{2}}f\left(\eta \right)\text{,}$$

Using Eqs. (10) and (11) in (1), (2), (3), (4), (5), (6), Eq. (1) satisfied and the other governing equations reduced as:

(12) $$f^{\prime \prime \prime }+Wef^{\prime \prime }f^{\prime \prime \prime }-{\left(f^{\prime }\right)}^{2}-ff^{\prime \prime }-S\left(f^{\prime }+\frac{\eta }{2}f^{\prime \prime }\right)-Mf^{\prime }-kf^{\prime }=0\text{,}$$

(13) $$\left(1+Rd\right)\theta^{\prime \prime }-\mathrm{Pr}\left(\frac{S}{2}\left(3\theta +\eta \theta^{\prime }\right)+2f^{\prime }\theta -\theta^{\prime }f\right)=0\text{,}$$

(14) $$\begin{array}{l} f^{\prime }\left(0\right)=1,\;f\left(0\right)=0,\;\theta \left(0\right)=1,\\ f^{\prime \prime }\left(\beta \right)=0,\;\theta^{\prime }\left(\beta \right)=0,\\ f\left(\beta \right)=\frac{S\beta }{2} \end{array}$$

After interpretation we obtained the following physical parameters as:

(15) $$\begin{array}{l} \;\text{S}=\frac{a}{b}\text{,}\mathrm{Pr}=\frac{\rho \upsilon c_{p}}{k}=\frac{\mu c_{p}}{k}\text{,}\;\text{Rd}=\frac{4\sigma T_{s}^{3}}{kk^{*}}\text{,}\;We=\Gamma x\sqrt{\frac{2b^{3}}{\upsilon {\left(1-at\right)}^{3}}},\;\\ M=\frac{\rho B_{0}^{2}}{\rho b}\left(1-at\right),\;k==\frac{\upsilon \phi }{kb}\left(1-at\right) \end{array}$$

Here in Eq. (15) Pr signifies the Prandtl number, S used for unsteadiness Parameter, Rd represents the radiation parameter and We is a fluid material constant, M is magnetic parameter, k represents porosity parameter and all of these are defined respectively. The Skin friction is defined as

(16) $${\text{C}}_{f}=\frac{{\left(S_{xy}\right)}_{y=0}}{\rho U_{w}^{2}},$$

Where $S_{xy}$ in Eq. (16) is defined as

(17) $$S_{xy}=\mu_{0}\left(\frac{\partial u}{\partial y}+\frac{\Gamma }{\sqrt{2}}{\left(\frac{\partial u}{\partial y}\right)}^{2}\right)=0$$

The dimensionless form of Eq. (17)

(18) $${\text{C}}_{f}\sqrt{{\text{Re}}_{x}}=f^{\prime \prime }\left(0\right)+W_{e}{\left(f^{\prime \prime }\right)}^{2}\left(0\right).$$

Where ${{\text{R}}_{\text{e}}}_{x}$ in Eq. (18) is called local Reynolds number. The Nusselt number is defined as $Νu=\frac{\delta Q_{w}}{\hat{k}\left(T-T_{0}\right)},$ in which $Q_{w}$ is the heat flux, where $Q_{w}=-\hat{k}{\left(\frac{\partial T}{\partial y}\right)}_{\eta =0}$, Here the dimensionless form of Νu is obtained in Eq. (19) below

(19) $$Νu=-\left(1+\frac{4}{3}Rd\right)\Theta^{\prime }\left(0\right),$$

## 3 Methodology

For solution of the problem we implement the Homotopy Analysis Method to fin the solution of Eqs. (12) and (13), consistent with the boundary constraints (14). The solutions enclosed the secondary parameters ℏ, which standardize and switches to the combination of the solutions. Initial solution of Eqs. (12) and (13) are given in Eq. (20)

(20) $$f_{0}\left(\eta \right)=\eta ,\;\theta_{0}\left(\eta \right)=1\text{,}$$

The linear operators can be chosen as

(21) $$L_{f}\left(f\right)=\frac{d^{4}f}{d\eta^{4}},\;{\text{L}}_{\theta }\left(\theta \right)=\frac{d^{2}\theta }{d\eta^{2}}\text{.}$$

The differential operators in (21) content are defined as

(22) $$\begin{array}{l} L_{f}\left(\psi_{1}+\psi_{2}\eta +\psi_{3}\eta^{2}+\psi_{4}\eta^{3}\right)=0,\\ L_{\theta }\left(\psi_{5}+\psi_{6}\eta \right)=0\text{.} \end{array}$$

Here in Eq. (22) $\sum_{i=1}^{6}\psi_{i},$ where i=1,2,3... are arbitrary constants. Expressing q∈[01] as an entrenching parameter with associate parameters $\hbar_{f}$ and $\hbar_{\theta }$ where ℏ≠0. Then the problem in case of zero order deform to the following form

(23) $$\left(1-q\right)L_{f}\left(\hat{f}\left(\eta ,q\right)-f_{0}\left(q\right)\right)=q\hbar_{f}{Ν}_{f}\left(\hat{f}\left(\eta ,q\right),\hat{g}\left(\eta ,q\right)\right),$$

(24) $$\left(1-q\right)L_{\theta }\left(\hat{\theta }\left(\eta ,q\right)-\theta_{0}\left(\eta \right)\right)=q\hbar_{\theta }{Ν}_{\theta }\left(\hat{f}\left(\eta ,q\right),\hat{g}\left(\eta ,q\right),\hat{\theta }\left(\eta ,q\right)\right).$$

The subjected boundary conditions for Eqs. (23) and (24) are obtained in Eq. (25)

(25) $$\begin{array}{l} f\left(0\right)=0,\;f^{\prime }\left(0\right)=1,\;f\left(\beta \right)=\frac{S\beta }{2},\;f^{\prime \prime }\left(\beta \right)=0,\\ \theta \left(0\right)=\theta^{\prime }\left(\beta \right)=0. \end{array}$$

The resultant nonlinear operators are

(26) $$\begin{array}{l} {Ν}_{f}\left(f\left(\eta ;q\right),\hat{\theta }\left(\eta ;q\right)\right)=f_{\eta \eta \eta }+Weff_{\eta \eta }-f_{\eta }f_{\eta }-ff_{\eta \eta }\\ -S\left[f_{\eta }+\frac{\eta }{2}f_{\eta \eta }\right]-Mf_{\eta }-kf_{\eta }\text{,} \end{array}$$

Using the Taylor's series expansion to expand f(η;q) and θ(η;q) in Eq. (26) in term of q we get

(27) $$\begin{array}{l} f\left(\eta ,q\right)=f_{0}\left(\eta \right)+\sum_{i=1}^{\infty }f_{i}\left(\eta \right),\\ \theta \left(\eta ,q\right)=\theta_{0}\left(\eta \right)+\sum_{i=1}^{\infty }\theta_{i}\left(\eta \right), \end{array}$$

Where

(28) $$f_{i}\left(\eta \right)={\frac{1}{i!}{\hat{f}}_{\eta }\left(\eta ,q\right)|}_{q=0},\;\theta_{i}\left(\eta \right)={\frac{1}{i!}{\hat{\theta }}_{\eta }\left(\eta ,q\right)|}_{q=0}\text{.}$$

Differentiating Zero^th^ order Eqs. (27) and (28) i^th^ time we obtained the i^th^ order deformation equations with respect to q dividing by i! and then inserting q=0. So *i*^*th*^ order deformation equations

(29) $$\begin{array}{l} L_{f}\left(f_{i}\left(\eta \right)-\xi_{i}f_{i-1}\left(\eta \right)\right)=h_{f}{ℜ}_{i}^{f}\left(\eta \right),\\ L_{\theta }\left(\theta_{i}\left(\eta \right)-\xi_{i}\theta_{i-1}\left(\eta \right)\right)=h_{\theta }{ℜ}_{i}^{\theta }\left(\eta \right)\text{.} \end{array}$$

The resultant boundary conditions for Eq. (29) are

(30) $$\begin{array}{l} f_{i}\left(0\right)=f_{i}^{\prime }\left(0\right)=f_{i}\left(\beta \right)=\frac{S\beta }{2},\;\;f_{i}^{\prime \prime }\left(\beta \right)=0,\\ \theta_{i}\left(0\right)=0,\theta_{i}\left(\beta \right). \end{array}$$

(31) $$R_{i}^{f}\left(\eta \right)=f_{i-1}^{\prime \prime \prime }\left(\eta \right)+We\sum_{j}^{i-1}f_{i-1-j}^{\prime \prime }f_{j}^{\prime \prime \prime }-\sum_{j=0}^{i-1}f_{i-1-j}^{\prime }f_{j}^{\prime }-f_{i-1-j}f_{j}^{\prime \prime }-S\left[f_{i-1}^{\prime }\left(\eta \right)+\frac{\eta }{2}f_{i-1}^{\prime \prime }\right]-Mf_{i-1}^{\prime }-kf_{i-1}^{\prime }\text{,}$$

(32) $$R_{k}^{\theta }\left(\eta \right)=\theta_{i-1}^{\prime \prime }\left(\eta \right)-\mathrm{Pr}\left[\sum_{j=0}^{i-1}f_{i-1-j}\theta_{j}^{\prime }-2\sum_{j=0}^{i-1}f_{i-1-j}^{\prime }\theta_{j}-\left(\frac{S}{2}\left(3\theta_{i-1}+\eta \theta_{i-1}^{\prime }\right)\right)\right].$$

Where

(33) $$\xi_{i}=\{\begin{array}{cc} 1, & \text{if}\;\;q>1\\ 0, & \text{if}\;q\leq 1 \end{array}$$

Hence (30), (31), (32), (33) are the final simplified equitation's.

## 4 Analysis

**Fig. 2:**
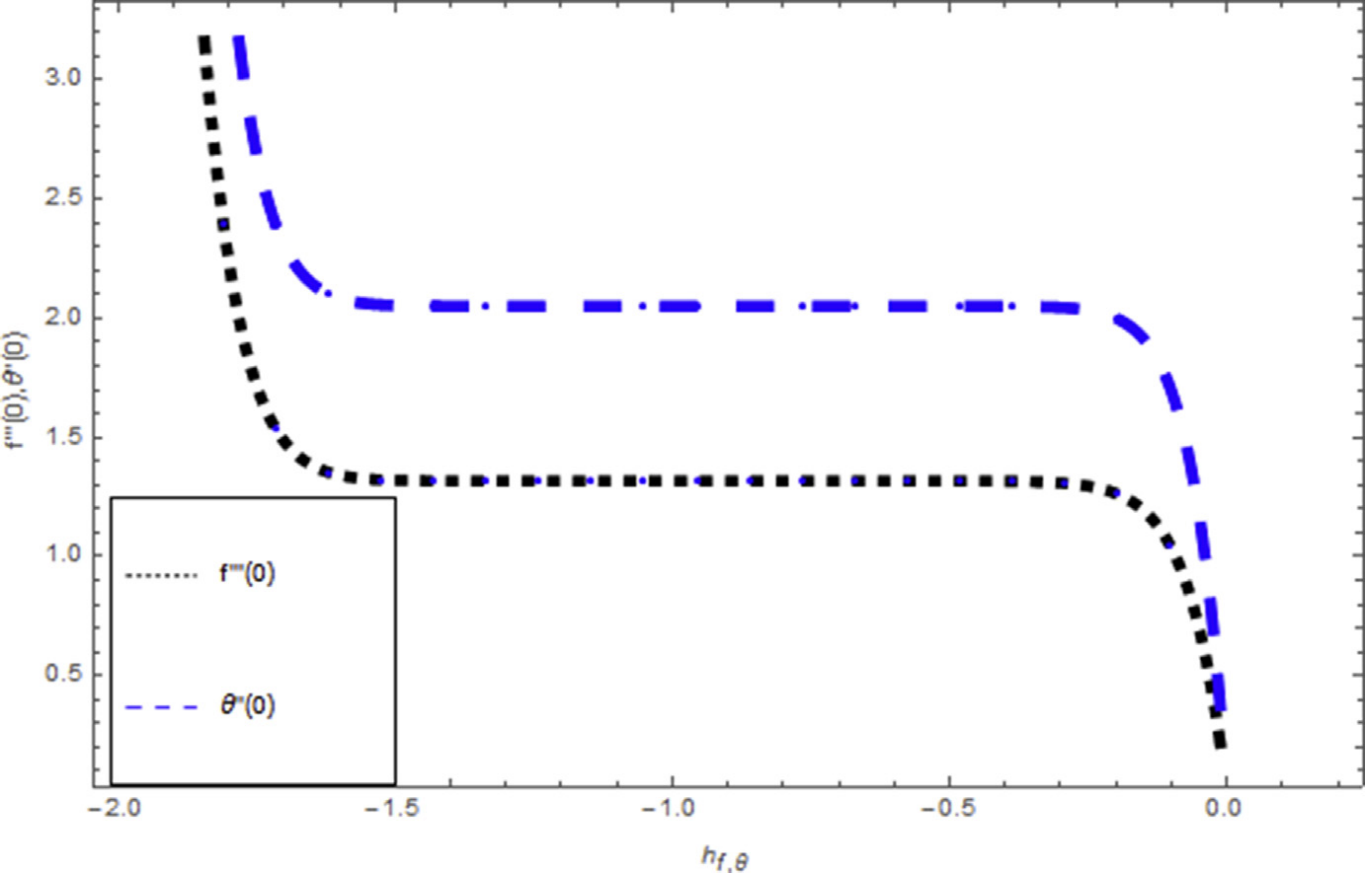
Combined h curves of f(η) and θ(η) at $12^{th}$ order approximation.

Here our interest is to analyze analytical solution of obtaining system of ordinary differential equations by Homotopy Analysis Method. When the series solution of the velocity and temperature profile are computed by HAM, the assisting parameters $h_{f},h_{\theta }$ seems which responsible for adjusting of convergence. In the acceptable region of h, h-curves of $f^{\prime \prime }\left(0\right)\;$ and $\theta^{\prime }\left(0\right)$ are plotted in Fig. 2, displaying the valid region. Table 1 displays the numerical values of HAM solutions showing that homotopy analysis technique is a speedily convergent technique.

**Table 1:** Convergence of $f^{\prime \prime }\left(0\right)\;and\;\;\Theta^{\prime }\text{(0)}\;$ by HAM method when We=0.2,β=Rd=Pr=S=k=0.1,M=0.5.

Solution Approximations	$f^{\prime \prime }\left(0\right)\;$	$\Theta^{\prime }\left(0\right)$
1	−1.90218	−0.24761
2	−1.90259	−0.214609
3	−1.90267	−0.219032
4	−1.90268	−0.218439
5	−1.90269	−0.218519
6	−1.90269	−0.218508
7	−1.90269	−0.218510
8	−1.90269	−0.218509
9	−1.90269	−0.218509

## 5 Results and discussion

**Fig. 3:**
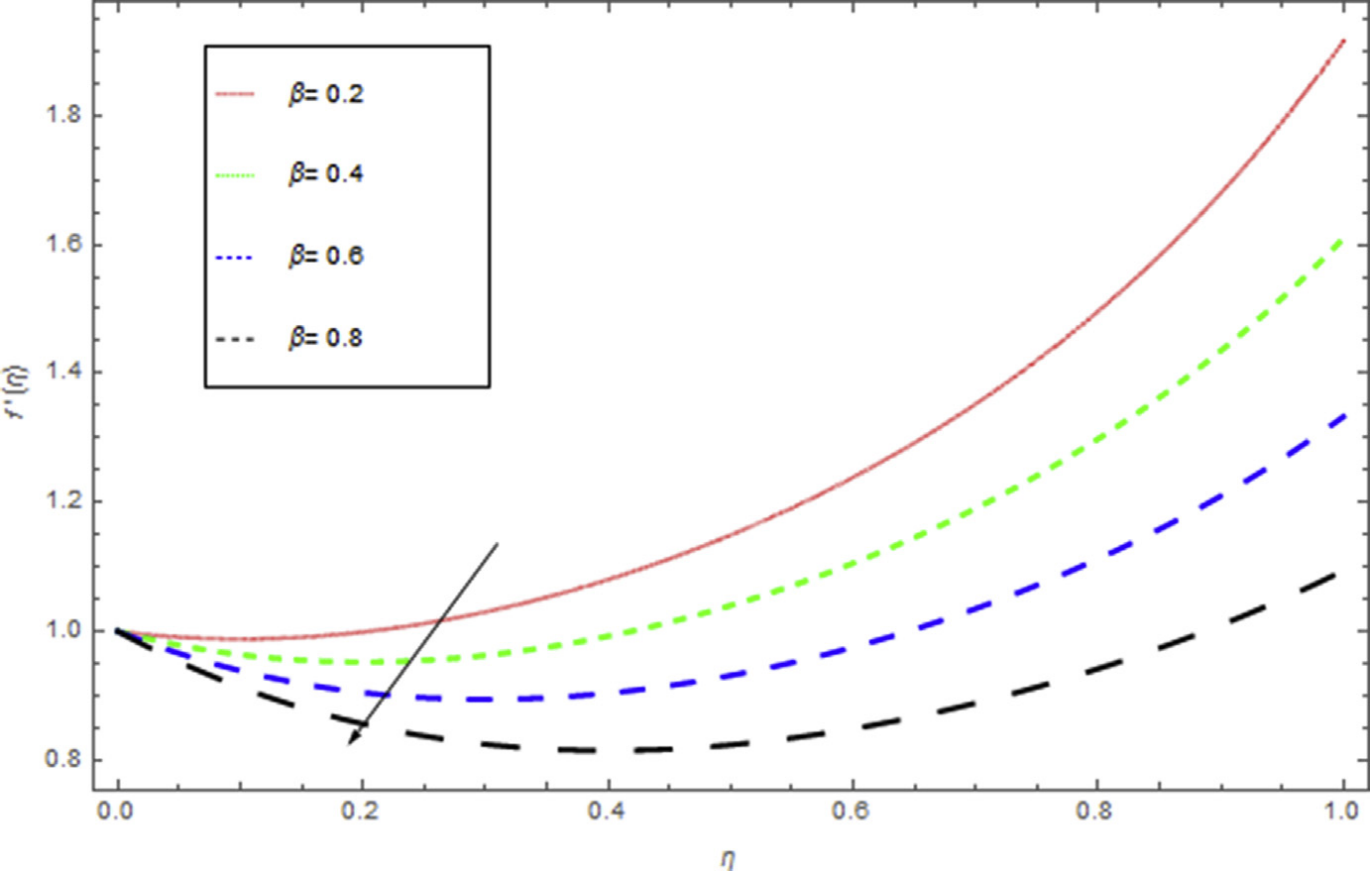
The influence of β on f(η) when h=−1.9,S=0.5,M=1,k=0.4,We=0.4.

**Fig. 4:**
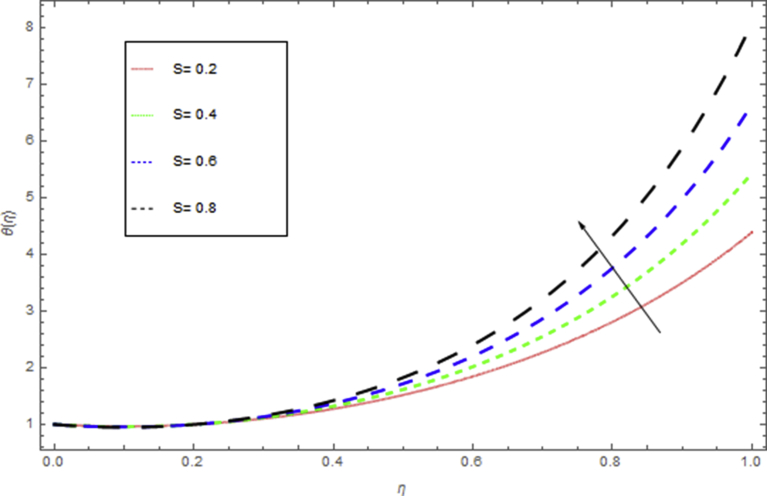
The influence of S on f(η) when h=1.2,β=We=0.5,k=0.4,M=1.

**Fig. 5:**
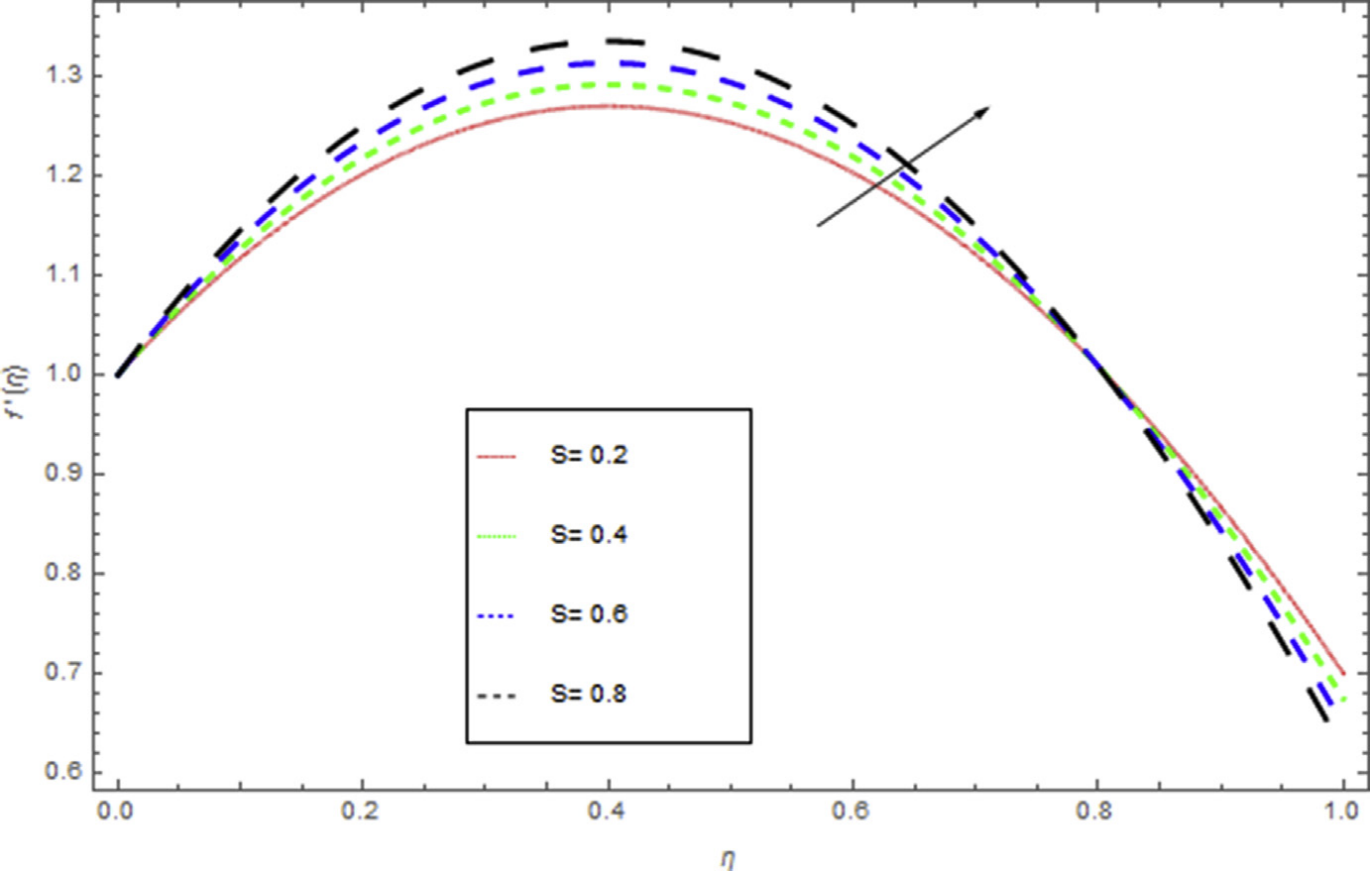
The influence of S on θ(η) when Rd=0.3,h=1.5,β=0.1,We=0.5,k=0.4,M=1,Pr=0.8.

**Fig. 6:**
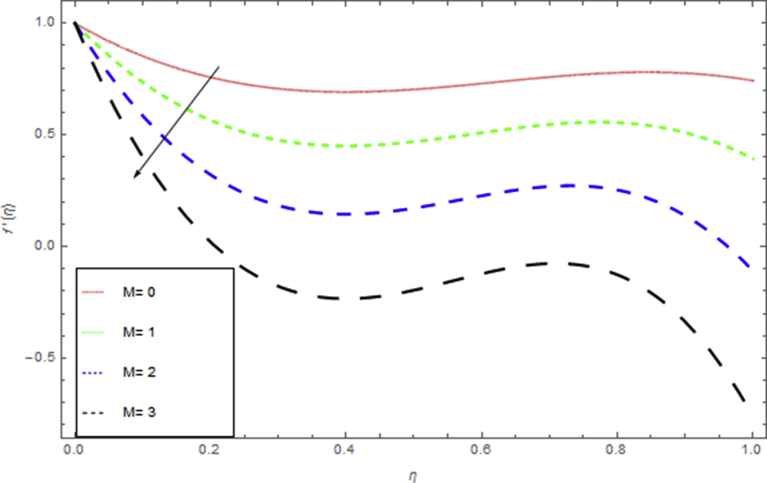
The influence of M on f(η) when h=1.3,β=We=0.5,k=0.4,S=0.2.

**Fig. 7:**
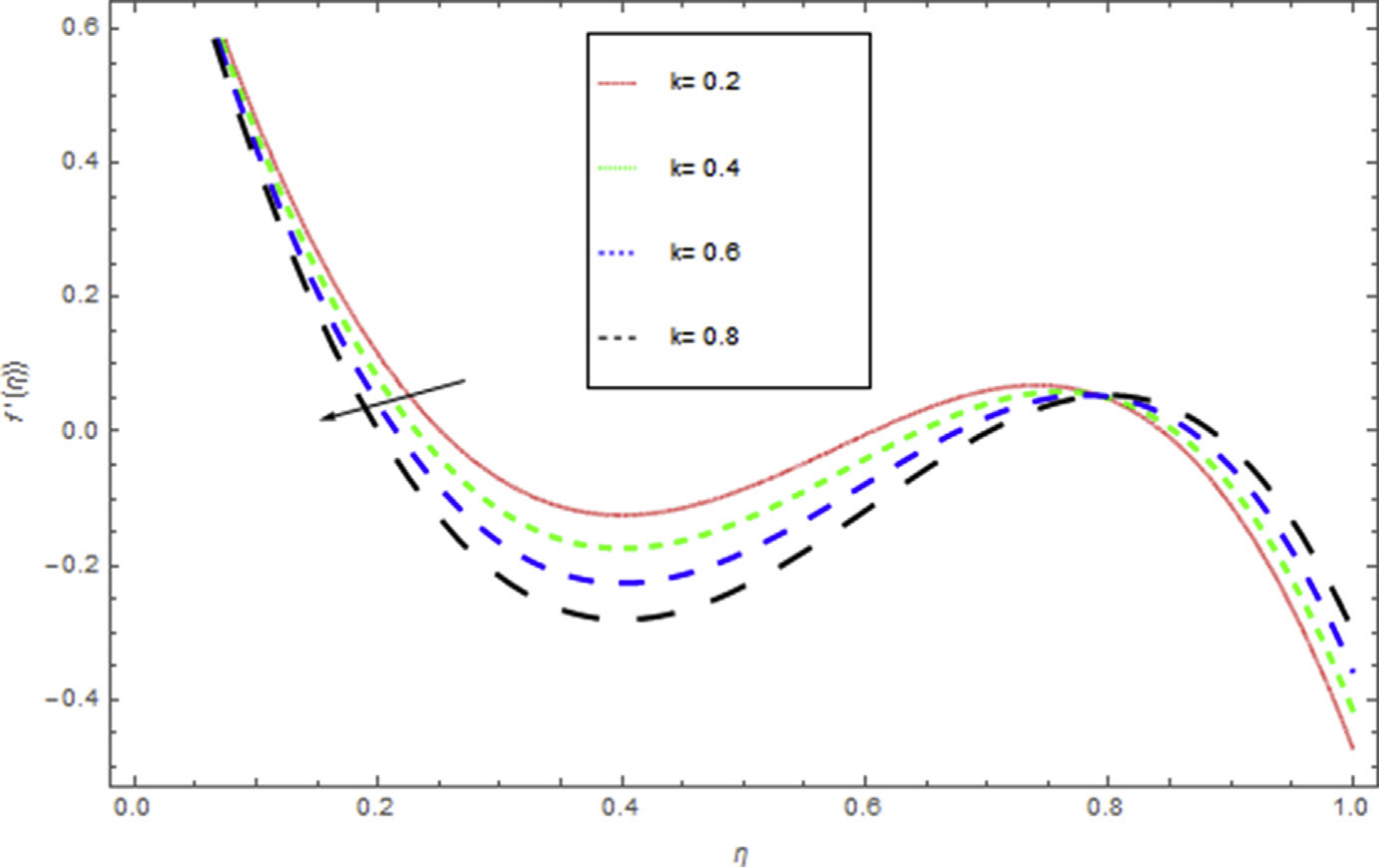
The influence of k on f(η) when h=0.9,β=0.4,We=0.6,S=0.4,M=1.

**Fig. 8:**
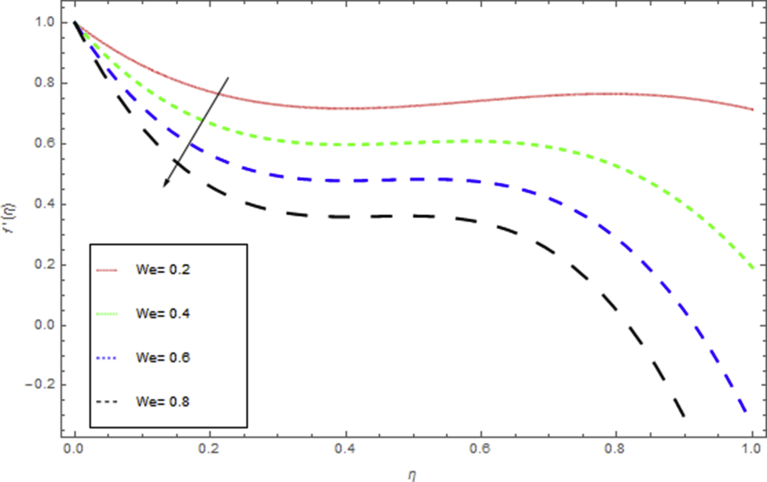
The influence of We on f(η) when h=−1.2,β=S=0.5,k=0.4,M=0.6.

**Fig. 9:**
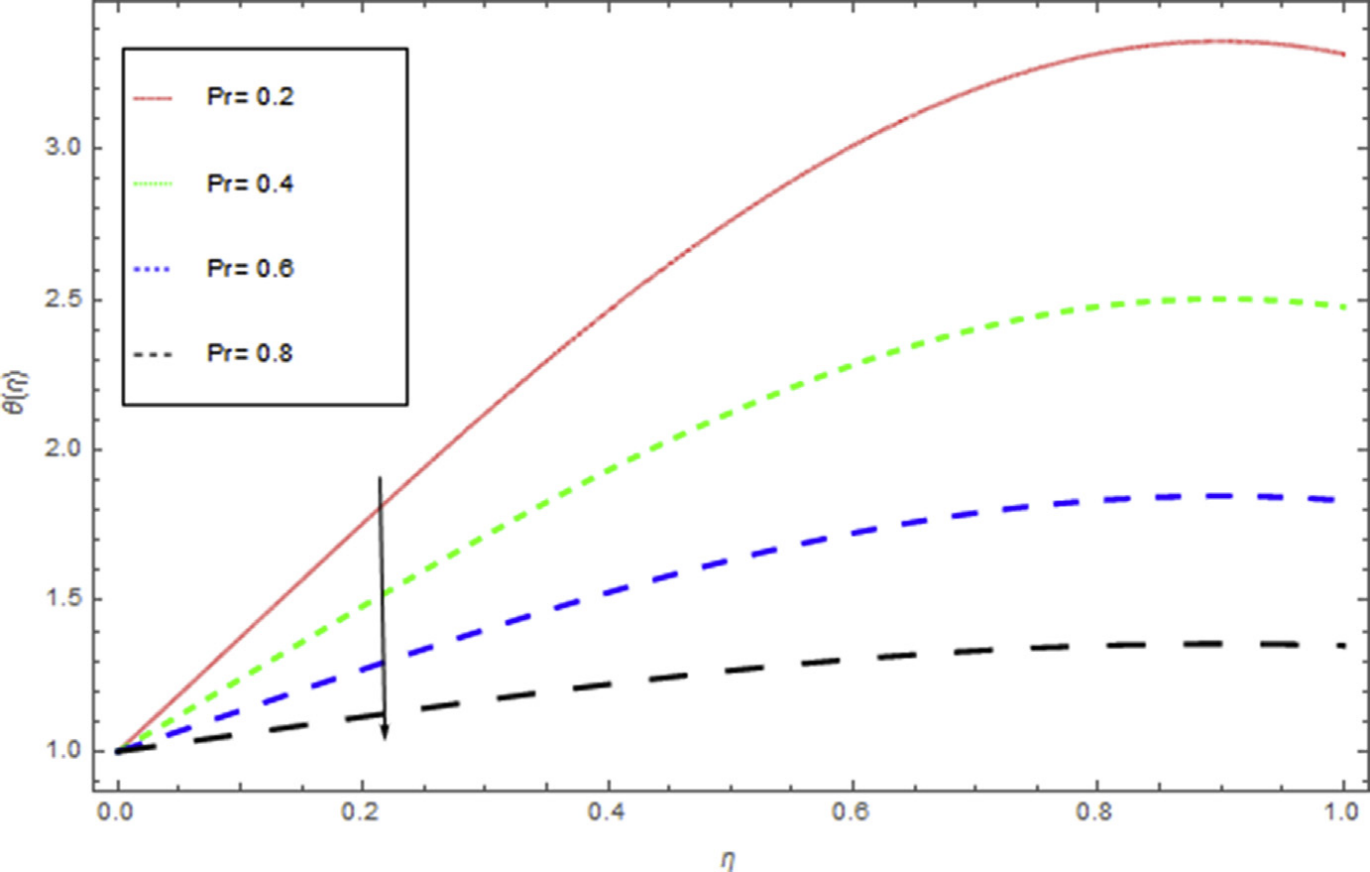
The influence of Pr on θ(η) when Rd=0.5,h=−1.5,β=0.4,We=0.5,k=0.6,M=0.5,S=0.4.

**Fig. 10:**
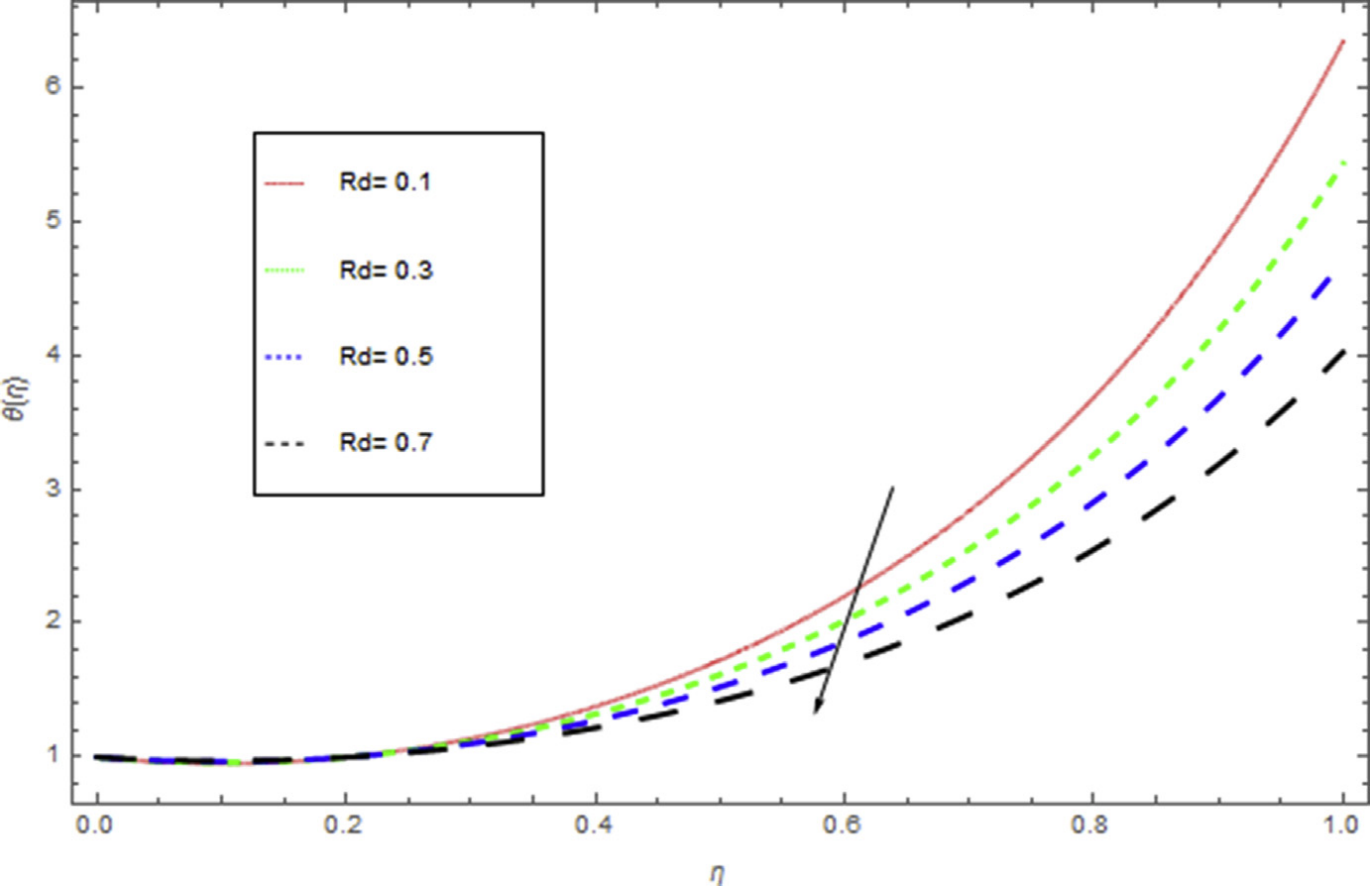
The influence of Rd on θ(η) when Pr=0.5,h=−1.5,β=0.4,We=0.5,k=0.6,M=0.5,S=0.4.

The current research has been conceded out to study the flow of Williamson liquid film flow in a time dependent starching sheet with the impact of MHD and thermal radiation. The determination of this section is to examine the physical consequences of different embedding parameters on the velocity f(η) and temperature Θ(η) profiles, which are illustrated in Figs. 3, 4, 5, 6, 7, 8, 9, and 10. Fig. 3 demonstrates the influence of the liquid film thickness *β* throughout the flow motion. Increasing β decreases the flow velocity of the liquid film. Actually fluid film produces opposition to the film flow and reduces f(η) with higher values of β. Fig. 4 determines the behaviour of the parameter S on the f(η). It is perceived that f(η) directly varies with unsteadiness parameter S. Increasing S rise the fluid motion. It is perceived that solution depended on the unsteadiness parameter S, the solution exist only when $S\in \left[\begin{array}{cc} 0 & 0 \end{array}\right]$. Fig. 5 shows the influence of the unsteadiness parameter S on the θ(η). It is perceived that θ(η) directly varies with unsteadiness parameter S. Increasing S rises the temperature and as a result increase the kinetic energy of the fluid, so the liquid film motion increases. Fig. 6 describes the characteristics of the magnetic strength M. When the magnetic strength rises on the sheet surface through the fluid flow, the internal fluid resistance increases, which causes reduction in the velocity field. The reason for this phenomenon is the enhancement of magnetic field to a fluid which crops a conflict force called the Lorentz force. This force carries the reduction in the motion of the fluid. Fig. 7 shows the impact of porosity parameter k on f(η), which has an domineering eccentric in the flow motion. It is perceived that the augmented value of k rises the porous space which makes resistance in the motion and reduces it speed. The motion of the fluid under the influence of We is deliberated in Fig. 8. The velocity f(η) decreases with rising values of We. In fact, increase in relaxation time produces resistance force and eventually declines the fluid velocity. The impact of Pr on Θ(η), is shown in Fig. 9. It is clear that temperature field declines with higher numbers of Pr and increases for small values of Pr*.* Fig. 10 shows the influence of radiation parameter Rd on temperature profile. When we increase thermal radiation parameter Rd, then it is perceived that it augments the temperature in the fluid layer. This increase leads to drop in the rate of cooling for thin film flow.

The numerical values of the surface temperature θ(β) for different value of M,Rd and k are given in Table 2. It is observed that the increasing values of M,Rd and k increase the surface temperature θ(β), where opposite effect is found for Pr, that is the large value of Pr reduces the surface temperature θ(β). The numerical values of the heat flux $\Theta^{\prime }\left(0\right)$ for dissimilar values of embedded parameters Rd,β,Pr,S have been shown in Table 3. It is perceived that larger values of thermal radiation Rd,β and Pr decrease the wall temperature and S increases the wall temperature gradient $\Theta^{\prime }\left(0\right)$. The numerical values of M,k,β and We on skin friction $C_{f}$ are given in Table 4. From this table it is obvious that high values of M,k and β decrease $C_{f}$ while increasing We increases skin friction.

**Table 2:** The Skin friction coefficient for dissimilar values of Re,Κr,β and γ when S=0.4.

M	Pr	Rd	k	Tawade et al [1] results θ(β)	Present results θ(β)
0.0	0.1	1.0	0.1	0.257696	0.223456
1.0				0.420739	0.432111
2.0				0.526782	0.712351
5.0				0.695757	1.023001
1.0	0.01			1.030899	1.625341
	0.1			0.931433	1.236540
	1.0			0.420739	0.988872
	5.0			0.011137	0.566100
	1.0	0.0		0.227566	0.222109
		1.0		0.420739	0.432091
		3.0		0.715871	0.674109
		5.0		0.826899	0.992221
		1.0	0.1	0.190930	0.011236
			0.2	0.223926	0.227634
			0.3	0.250515	0.537000
			0.4	0.281804	0.719273
				0.340312	1.200235

**Table 3:** Wall temperature gradient $\Theta^{\prime }\left(0\right)$ verses various value of embedded parameters when h=0.1.

Rd	β	Pr	S	$\Theta^{\prime }\left(0\right)$
0.0	0.2	1.0	0.2	0.682385
0.5				0.541422
1.0				0.440569
2.0				0.311380
1.0	0.1			0.411411
	0.2			0.321022
	0.3			0.300420
	0.4			0.291420
	0.5			0.111427
	0.1	0.1		0.411420
		0.5		0.371420
		1.5		0.182285
		5.0		0.011422
		10		0.000569
		1.0	0.2	0.411420
			0.4	0.612427
			0.6	0.891428
			0.8	1.500987
			1.0	2.087651

**Table 4:** The effects of dissimilar values of M,k,β and We on Skin friction coefficient.

M	k	β	We	−(CfRex)12
0.1	0.5	1.0	1.5	3.33027
0.5				2.94882
1.0				2.64208
1.5	0.1			4.33999
	0.5			4.32157
	1.0			4.26897
	1.5	0.1		5.64227
		0.5		5.44576
		1.0		4.89911
		1.5	0.1	4.12743
			0.5	4.35772
			1.0	5.13048
			1.5	5.91612

## 6 Conclusion

The conclusion of the present work is mainly focused on the behaviour of embedded parameters and solutions of the obtained results. The central concluded points are:

• Thermal boundary layer thickness reduces with rise of radiation parameter Rd So, Nusselt number Νu rises with rise of radiation parameter Rd.
• The increasing values of M,Rd and k increase the surface temperature θ(β), where opposite effect is found for Pr, that is the large values of Pr reduce the surface temperature θ(β).
• Increasing k reduce the flow of thin films.
• For skin friction $C_{f}$ it is found that it increases when the viscosity parameter R is decreased.
• It is notice that the strong magnetic field reduce the velocity he liquid films.
• It is also concluded that liquid film flow is affected by the Lorentz's force.

## Declarations

### Author contribution statement

Zahir Shah, Ebenezer Bonyah, Saeed Islam, Waris Khan, Mohammad Ishaq: Conceived and designed the analysis; Analyzed and interpreted the data; Contributed analysis tools or data; Wrote the paper.

### Funding statement

This research did not receive any specific grant from funding agencies in the public, commercial, or not-for-profit sectors.

### Competing interest statement

The authors declare no conflict of interest.

### Additional information

No additional information is available for this paper.
